# Molecular dynamics simulation studies and *in vitro *site directed mutagenesis of avian beta-defensin Apl_AvBD2

**DOI:** 10.1186/1471-2105-11-S1-S7

**Published:** 2010-01-18

**Authors:** Soja Saghar Soman, Krishnankutty Chandrika Sivakumar, Easwaran Sreekumar

**Affiliations:** 1 Molecular Virology Laboratory, Department of Molecular Microbiology, Rajiv Gandhi Centre for Biotechnology (RGCB), Thycaud PO, Thiruvananthapuram-695014, Kerala, India; 2 Bioinformatics facility, Rajiv Gandhi Centre for Biotechnology (RGCB), Thycaud PO, Thiruvananthapuram-695014, Kerala, India

## Abstract

**Background:**

Defensins comprise a group of antimicrobial peptides, widely recognized as important elements of the innate immune system in both animals and plants. Cationicity, rather than the secondary structure, is believed to be the major factor defining the antimicrobial activity of defensins. To test this hypothesis and to improve the activity of the newly identified avian β-defensin Apl_AvBD2 by enhancing the cationicity, we performed *in silico *site directed mutagenesis, keeping the predicted secondary structure intact. Molecular dynamics (MD) simulation studies were done to predict the activity. Mutant proteins were made by *in vitro *site directed mutagenesis and recombinant protein expression, and tested for antimicrobial activity to confirm the results obtained in MD simulation analysis.

**Results:**

MD simulation revealed subtle, but critical, structural variations between the wild type Apl_AvBD2 and the more cationic *in silico *mutants, which were not detected in the initial structural prediction by homology modelling. The C-terminal cationic 'claw' region, important in antimicrobial activity, which was intact in the wild type, showed changes in shape and orientation in all the mutant peptides. Mutant peptides also showed increased solvent accessible surface area and more number of hydrogen bonds with the surrounding water molecules. In functional studies, the *Escherichia coli *expressed, purified recombinant mutant proteins showed total loss of antimicrobial activity compared to the wild type protein.

**Conclusion:**

The study revealed that cationicity alone is not the determining factor in the microbicidal activity of antimicrobial peptides. Factors affecting the molecular dynamics such as hydrophobicity, electrostatic interactions and the potential for oligomerization may also play fundamental roles. It points to the usefulness of MD simulation studies in successful engineering of antimicrobial peptides for improved activity and other desirable functions.

## Background

Defensins are recognized as important elements of the innate immune system in almost all living beings [1]. The most distinct molecular feature of defensins is their high pI value, ranging from +6 to +12 as monomers, manifested by abundant arginine and lysine residues in their sequences [2]. They kill microorganisms through permeabilization of the microbial membrane composed of negatively charged components such as phospholipids, teichoic acids and lipopolysaccharides [3]. It is believed that electrostatic interactions dictate not only the uptake of cationic defensins across the bacterial cell wall but also their ability to permeabilize the cytoplasmic membrane and to induce leakage of cellular contents [4].

Many previous studies hypothesized that the major factor defining the antimicrobial activity of defensin is its cationicity or its isoelectric point (pI) value, than its secondary structure [5]. The present study attempted to verify this hypothesis and generate derivatives of a beta-defensin with more microbicidal activity by engineering the protein. Apl_AvBD2 is a β-defensin homologue identified from domestic duck, and was found to exhibit antibacterial and immunomodulatory properties [6,7]. In the present study, we made *in silico *mutants of Apl_AvBD2 with higher pI values than the wild type, and analyzed them by Molecular Dynamic (MD) simulation analysis to find the structure-function relationship. Subsequently, mutated recombinant proteins were made *in vitro *and were evaluated for antibacterial activity to confirm the observations from the computational studies. Our results indicated that subtle structural differences in critical areas of the molecule can drastically alter the antibacterial potential of β-defensin molecules.

## Methods

### Homology modelling of wild type and *in silico *mutated Apl_AvBD2 peptides

The predicted amino acid sequence of Apl_AvBD2 [GenBank: AY641439] was subjected to a homology search using BLAST ([8] and PSI-BLAST [9] against NCBI PDB database. The top hits were aligned against the query sequence in a multiple sequence alignment using Clustal W [10]. We chose the top scoring sequence Apa_AvBD2 (Spheniscin 2: showed 35% sequence identity), originating from King Penguin as the template for further study and the PDB co-ordinate ((PDB id: 1ut3) was retrieved from Protein Data Bank. Homology modelling was carried out using MODELLER package [11] based on the sequence alignment generated between template and target sequences. The atomic coordinates were obtained from the template structures to model Apl_AvBD2. Care was taken to make the coordination geometry of side chain atoms most favorable. Conformations of a few residues were therefore adjusted using loop refinement programme within MODELLER package. Energy minimization of the top scored model was carried out with GROMACS 3.3.1 (The Groningen Machine for Chemical Simulations) [12] using OPLSAA force field. The minimization was set to run for 5000 steps or until convergence to machine precision. PROSA2003 [13] program was used for validation of the model, by analyzing residue interaction energy and z-score. These procedures were iterated several times until a good quality model was obtained.

**Figure 1:**
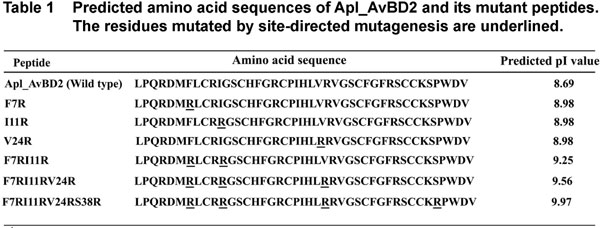
**Predicted amino acid sequences of Apl_AvBD2 and its mutant peptides**. The residues mutated by site-directed mutagenesis are underlined.

The dimeric state of Apl_AvBD2 protein was generated using SymmDock [14], an algorithm for the prediction of complexes with symmetry by geometry based docking. The structural representations of Apl_AvBD2 monomers and dimers were analyzed and visualized using PyMol software [15]. Based on the structure of a dimeric complex of Apl_AvBD2 created by the software, the homology models of the *in silico *mutants were made. Less cationic residues in several positions of the wild type protein were initially mutated to more cationic arginine residues. However, it was found that many of them changed the predicted structure of the protein. So only those mutants, which had the native predicted structure (Figure 1), were selected for further analysis.

### Molecular dynamics (MD) simulation of Apl_AvBD2 and Its mutant peptides

Molecular dynamics (MD) simulations were performed using the GROMACS version 3.3.1 and OPLSAA force field. The initial structures were solvated with three-point transferable intermolecular potential (TIP3P) water molecules [16] and appropriate number of Chloride ions in a rectangular box to neutralize the system; the box dimensions ensured that any protein atom was at least 8 Å away from the wall of the box. After energy minimization, MD simulations were performed for 100 ps at constant temperature (300 K) and pressure (1 atm) with periodic boundary conditions, particle-mesh Ewald summation, and a 1-fs time step to heat and equilibrate the system. This was followed by production runs of 10 ns duration for each simulation. Structures were saved every 10 ps for analysis. The relative binding energies computed using the tool g_energy module of 'GROMACS 3.3.1, employing molecular mechanics and a continuum solvent model. The output files (.xvg) from the GROMACS 3.3.1 was analysed in XMGRACE [17] software.

The parameters analysed were: area per atom, area per residue, energy variations (kinetic energy, potential energy and total energy), van der Waals interactions, intra-molecular hydrogen bonds, inter-molecular hydrogen bonds with surrounding water molecules, radius of gyration, root mean square deviations (RMSD) of each amino acid residue, root mean square fluctuations (RMSF) of Cα atoms of each amino acid residue, solvent accessible surface area, and hydrophobic and hydrophilic interactions. Pressure, volume, temperature, minimum distance to periodic image and maximum internal distance of the MD simulation system were examined. The obtained parameters for wild type and mutant peptides were compared.

### *In vitro *site directed mutagenesis and evaluation of antibacterial activity of wild type and mutant Apl_AvBD2

Mutagenesis reactions were carried out using the modified mutagenic primers as per the protocol by Quick Change Mutagenesis Kit (Stratagene, La Jolla, CA). Apl_AvBD2-pET-32 gene-vector construct was used for this purpose. The cloning strategy and recombinant expression protocols are described elsewhere [6]. Selected clones were sequenced directly using an ABI 3730 Genetic Analyzer automated DNA sequencer (PE Applied Biosystems, Foster City, CA) for confirmation of mutation. Recombinant proteins of single amino acid mutants and serial progressive mutants of Apl_AvBD2, which were selected for the MD analysis (Figure 1), were made *in vitro. *These would make variants of the protein with pI value ranging from 8.69 to 9.97. These proteins were expressed in BL21DE3 pLysS bacterial cells, purified and used for antibacterial assay [6].

## Results and discussion

### Homology modelling

Apl_AvBD2 was modelled using the known three-dimensional structure of the Apa_AvBD2 as the template for homology modelling. Superimposition of the Cα atoms of the template and target protein gave a calculated RMSD of 0.39 Å. Analysis of the secondary structure revealed the presence of mainly two β-strands in Apl_AvBD2. PROSA2003 Z-scores pointed to the compatibility between the model and the template. The Z-scores of both the structures were more or less similar. PROSA2003 quality check showed that the model of the Apl_AvBD2 was of good quality, wherein the interaction energy of each residue with the residual of the protein was negative. The Apl_AvBD2 model had a Z-score of -5.55 compared to that of the template (-5.83). Inspection of PROSA2003 plots revealed no region of the model with positive PROSA2003 energies (data not shown).

### Molecular dynamics (MD) simulation

In the MD simulation experiments, we selected dimeric state of the Apl_AvBD2 and its mutants for analysis. This was because several previous studies have shown that the β-defensins function in the form of dimers, which are their most stable form [2]. We also observed that in MD simulation analysis using the monomer form of wild type Apl_AvBD2, the system was not stable after 5 ns, confirming the monomer's instability (data not shown).

**Figure 2:**
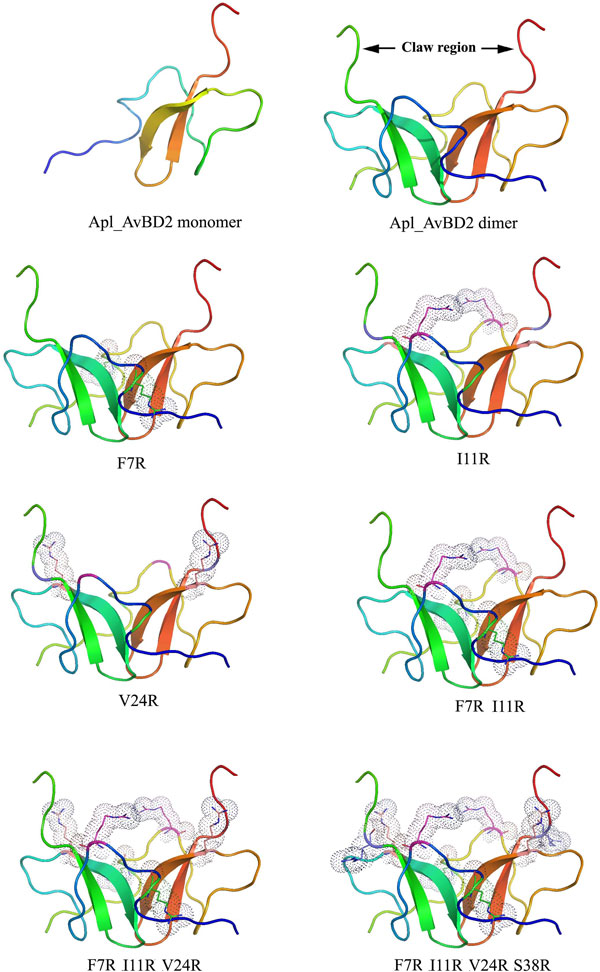
**Homology models of Apl_AvBD2 and its mutants**. The homology models revealed that the mutated amino acid residues were projected towards the 'claw' region (indicated by arrow) of the dimer and this may interfere its initial attachment with the bacterial cell membrane. The dotted area represents the substituted arginine residues.

**Figure 3:**
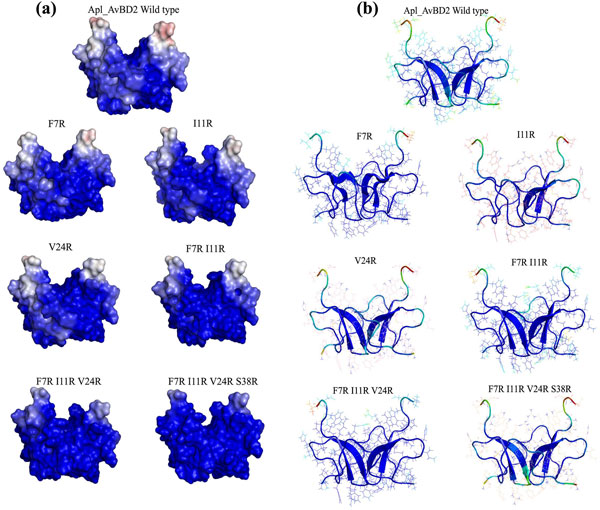
**Distribution of charged residues and flexibility of Apl_AvBD2 and its mutants during MD simulations**. (a) Distribution of charged residues on the solvent-accessible surfaces. Positively charged residues are represented as blue and negatively charged areas shown as red. (The potentials range from -5 *kT/e *for red to +5 *kT/e *for blue). As the number of arginine residues increased the cationicity of the peptide surface also increased. (b) Flexible regions in the average MD simulation structure of the peptides. The C-terminal residues and the loops of wild type, F7R, I11R and F7R I11R V24R S38R appeared to be more flexible. The dark blue areas represent the rigid regions and flexibility of the structure increases as dark blue turns light blue to red. The length of the β-sheets was varied in some of the mutants. The single amino acid mutant of Apl_AvBD2 (I11R) showed loss of two β-sheets in its structure.

In the analysis, the dimer models showed a distinct 'claw' region formed by the C-terminal tail region of each monomer (Figure 2). The substituted arginine residues appeared to be bulkier and projected to the C-terminal region of the peptides. The steric hindrances due to these arginine residues were found to be more pronounced in the mutant peptides. Increase in the number of arginine residues increased the cationicity of the peptide surface (Figure 3a). The C-terminal region of wild type peptide was more flexible compared to the mutants (Figure 3b). There were variations in the flexibility of different regions of the dimerized peptides as the number of arginine residues increased. The Root Mean Square Deviation (RMSD) and Root Mean Square Fluctuation (RMSF) were similar in the wild type and mutant type. A major change observed among the mutants was the loss of two β-sheets in the dimer complex of I11R peptide while simulation. The average simulation picture showed that the secondary structure of wild peptide was more stable compared to the mutants

**Figure 4:**
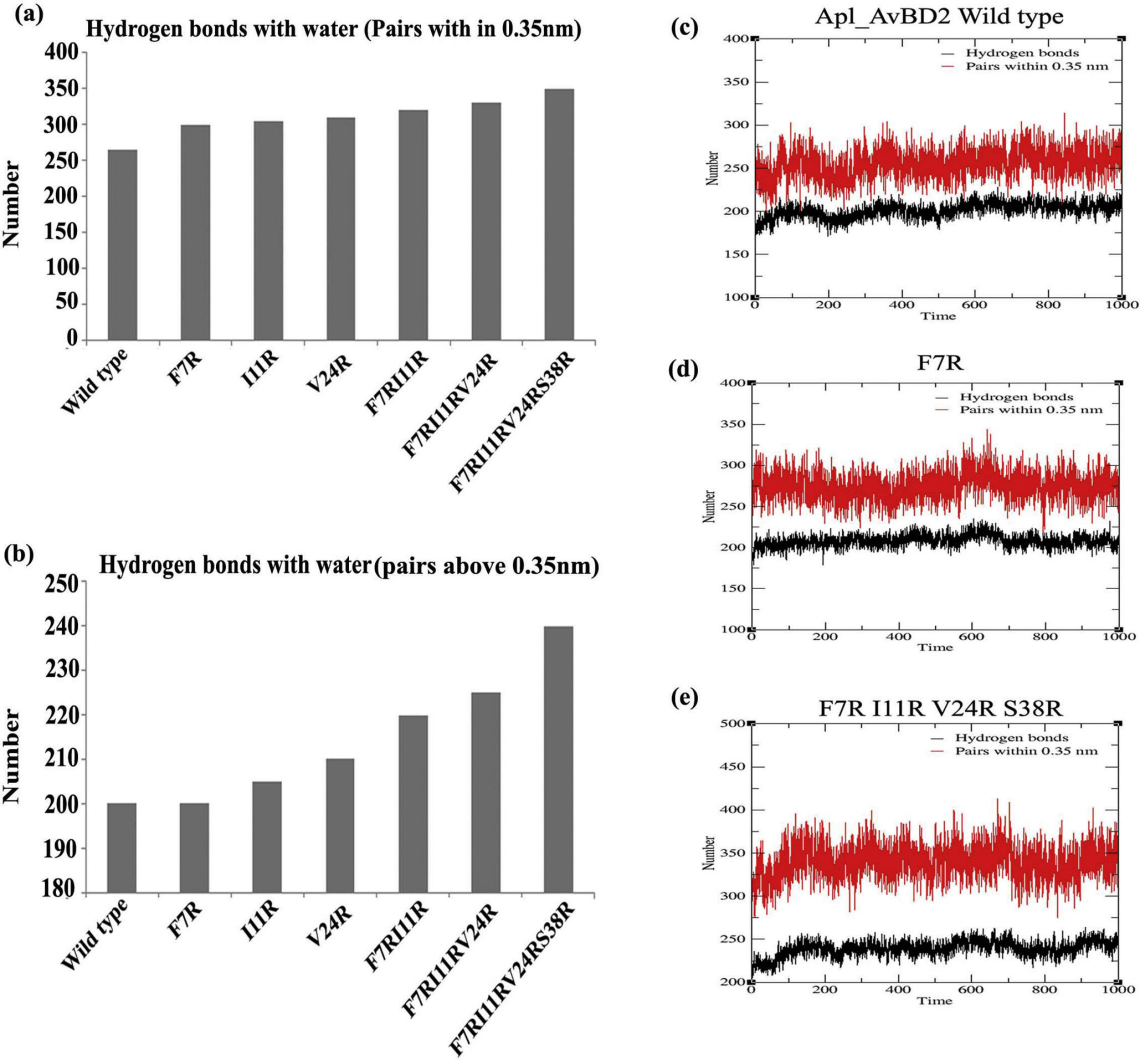
**Hydrogen bonds of Apl_AvBD2 and its mutants with water molecules**. (a) Hydrogen bond pairs within 0.35 nm and (b) Hydrogen bond pairs > 0.35 nm. The graph indicates average of values obtained during the entire period of simulation for each mutant. The representative original graphical data obtained for wild type, single mutant and progressive serial mutant are shown as (c), (d) & (e). As cationicity increases, the number of hydrogen bonds between peptide and the surrounding water molecules also increases. The change was visible with the substitution of a single amino acid itself.

**Figure 5:**
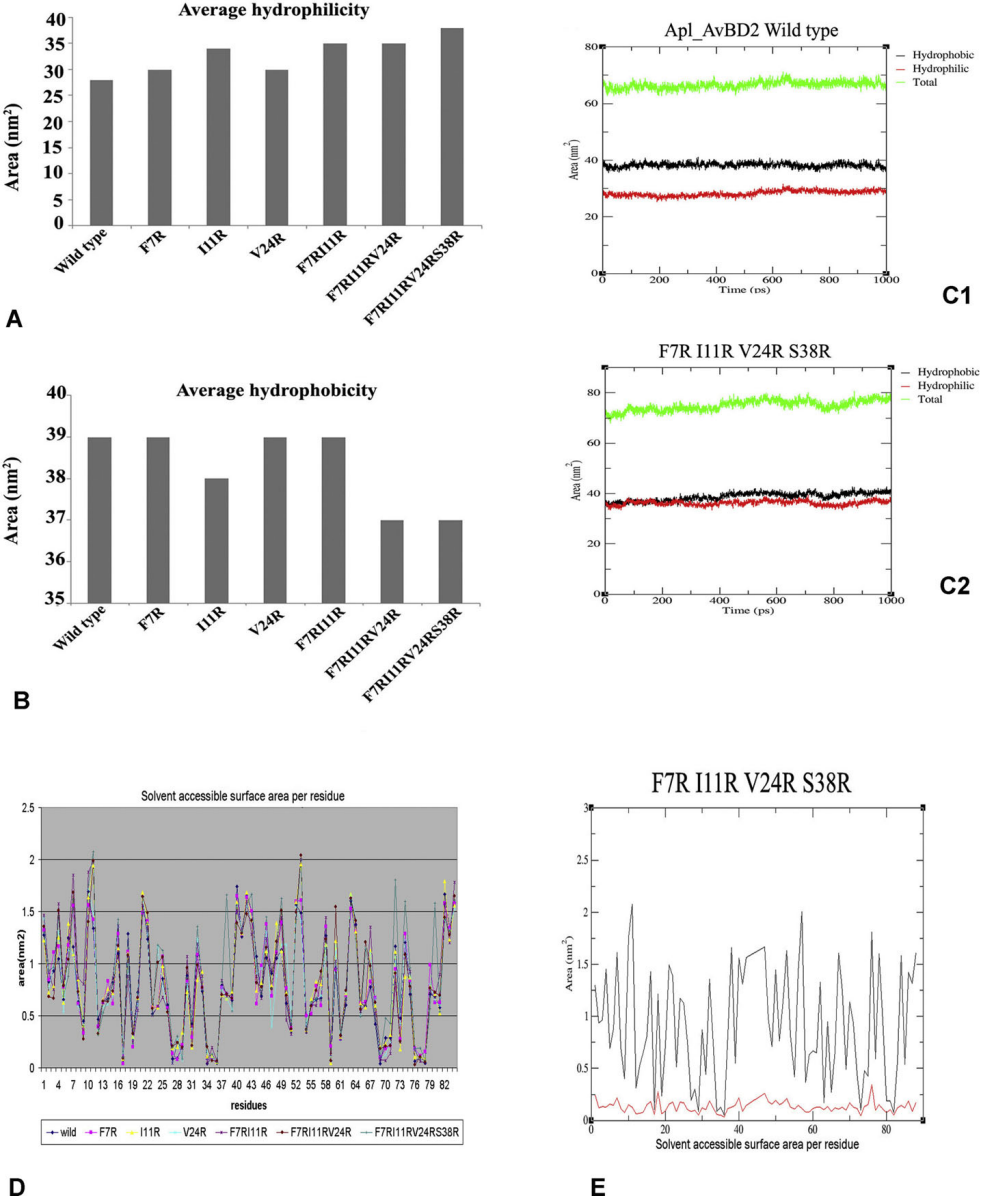
**Average hydrophilicity, hydrophobicity and solvent accessible surface area of Apl_AvBD2 and its mutants**. (A) Average Hydrophilicity, (B) Average hydrophobicity. The graph represents average of the values obtained during the entire period of simulation for each mutant. The representative original data obtained for wild type (C1) and one of the mutants (C2) are shown. (D) Solvent accessible surface area-merged figure of the data obtained for individual mutants. Original data for one of the mutants is also shown (E).

The number of hydrogen bonds between the peptide and the surrounding water molecules were more in mutants (Figure 4). As arginine residues increased, there was reduction in hydrophobicity and increase in solvent accessible surface area. This change was visible with the substitution of a single amino acid itself (Figure 5). It is reported that antimicrobial peptides need an optimal "hydrophobicity threshold" for insertion into zwitterionic micellar membranes [18] and hydrophobic interactions are necessary for the membrane 'sinking' process after the initial attachment [19]. Ideally, active antimicrobial peptides must have an appropriate balance of hydrophobicity and net positive charge [20]. The mutations introduced to the wild type Apl_AvBD2 were found to disturb this balance.

**Figure 6:**
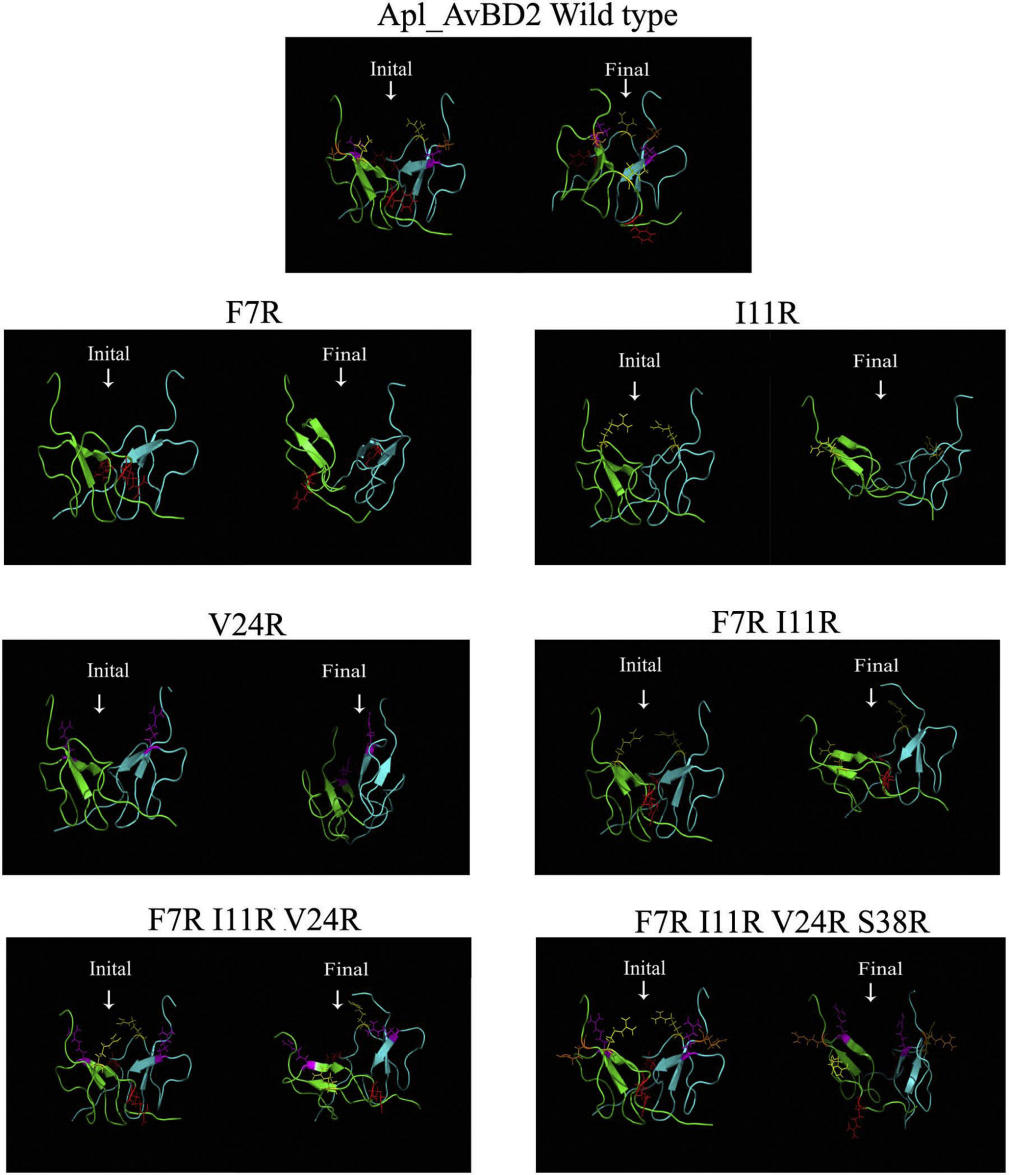
**Conformations Apl_AvBD2 and its mutants during the MD simulation experiments for 1000 ps**. The initial and final conformations are shown. Apl_AvBD2 form a stable dimer in the simulation system. The amino acids in the wild peptide are shown as sticks in different colours. The positions of amino acids in the mature peptide region selected for arginine substitution are represented as follows; red: 7^th ^position (Phenylalanine to Arginine), yellow: 11^th ^position (Isoleucine to Arginine), Magenta: 24^th ^position (Valine to Arginine), Orange: 38^th ^position (Serine to Arginine). The C-terminal residues form a distinct 'claw region (indicated by arrows) of the wild type Apl_AvBD2. In all the mutants, the shape and orientation of this claw region became less distinct during simulation.

Suresh and Verma [2] suggested the importance of the C-terminal 'claw' in the antibacterial activity of β-defensins. This region is supposed to act as a prehensile grasp to the bacterial cell membrane during the initial interaction. The shape and the orientation of C-terminal 'claw' region in the Apl_AvBD2 dimers were varied as time progresses (Figure 6). This structure was well formed and intact during the entire simulation period in the wild type peptide compared to the mutant types. This observation predicted an altered activity of the mutant peptides during functional assays.

### Functional evaluation of the antibacterial activity of wild type and mutant Apl_AvBD2

**Figure 7:**
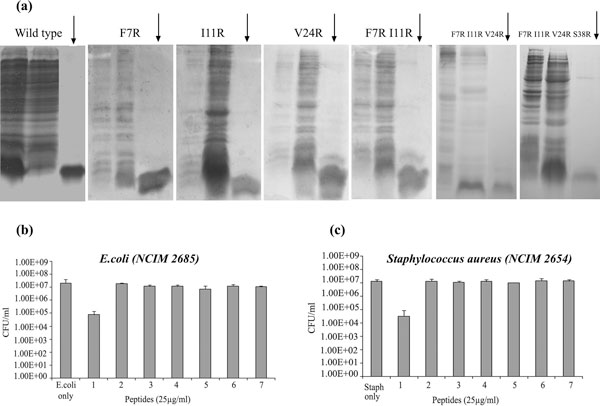
**Evaluation of antibacterial activity of wild type and mutant type Apl_AvBD2**. (a) SDS-PAGE of purified recombinant proteins (indicated by arrows). The recombinant wild Apl_AvBD2 showed antibacterial activity against the (b) Gram negative bacteria *E. coli (NCIM 2685) *and **(c) **Gram positive bacteria *Staphylococcus aureus (NCIM 2654) *in the standard plate count assay (SPC). The numbers in graphs represent: (1) wild type (2) F7R (3) I11R (4) V24R (5) F7R I11R (6) F7R I11R V24R (7) F7R I11R V24R S38R. Mutant proteins do not show antibacterial activity.

The recombinant wild type Apl_AvBD2 protein showed antibacterial activity against the Gram negative organism *E. coli *NCIM2685 and against the Gram positive *Staphylococcus aureus *NCIM2654 (Figure 7) in the standard plate count assay (CFU/mL). The protein brought about three-log reduction in the colony forming units (cfu) of these test microbes as evidenced by the assay. However, the mutant peptides exhibited complete loss of antimicrobial activity. The serial mutants and single amino acid mutants showed this loss of activity. This corroborated observations in our preliminary MD simulation analysis, wherein the mutants, which had an intact predicted secondary structure, showed differences during the dynamic state of simulation. This further confirms the previous observations [21] that though the primary structure is the most important determinant of the activity of an antimicrobial peptide, the number of hydrogen bonds, hydrophobicity, water solubility, electrostatic interactions, potential for oligomerization and secondary structures such as α-helix and β-sheets also play critical roles in its bactericidal activity.

## Conclusion

From the results obtained in this study, it can be concluded that the increase in cationicity alone may not enhance the antibacterial activity of defensins. The antimicrobial activity of these peptides requires a balance between its cationicity and hydrophobicity. The substitution of hydrophobic residues with more cationic hydrophilic residues leads to complete loss of activity. Moreover, even a single amino acid change can cause deleterious effect in the antibacterial activity. Most of the naturally occurring antimicrobial peptides have undergone evolutionary selection to perform best in the host. Manipulations to enhance its effects may not always yield viable results and has to be done prudently. MD simulation experiments would be useful while manipulating antimicrobial peptides to improve its activity by mutagenesis or by chemical synthesis.

## Competing interests

The authors declare that they have no competing interests.

## Authors' contributions

SSS and KCS carried out the sequence alignment, molecular modelling and simulations, and drafted the manuscript. SSS performed the *in vitro *mutagenesis, recombinant expression and antibacterial assays. ES and SSS conceived the study. ES supervised the study design, coordination and edited the manuscript. All three authors read and approved the final manuscript.
